# (2-Methyl­phen­yl)(phen­yl)methanol

**DOI:** 10.1107/S1600536810029417

**Published:** 2010-07-31

**Authors:** B. P. Siddaraju, H. S. Yathirajan, B. Narayana, Seik Weng Ng, Edward R. T. Tiekink

**Affiliations:** a Department of Chemistry, V. V. Puram College of Science, Bangalore 560 004, India; b Department of Studies in Chemistry, University of Mysore, Manasagangotri, Mysore 570 006, India; c Department of Studies in Chemistry, Mangalore University, Mangalagangotri, 574 199, India; d Department of Chemistry, University of Malaya, 50603 Kuala Lumpur, Malaysia

## Abstract

In the title compound, C_14_H_14_O, the two benzene rings are almost orthogonal [dihedral angle = 87.78 (8) °]. The hy­droxy group lies approximately in the plane of its attached benzene ring [O—C—C—C torsion angle = −17.47 (17)°], and the hydroxyl and methyl groups lie to the same side of the mol­ecule and are *gauche* to each other. In the crystal, a hexa­meric aggregate mediated by a ring of six O—H⋯O hydrogen bonds occurs, generating an *R* ^6^_6_(12) loop.

## Related literature

For general background to the use of benzhydrols, see: Ohkuma *et al.* (2000 ▶). For the use of the title compound in the perfume and pharmaceutical industries, see: Meguro *et al.* (1985 ▶). For related diphenyl­methanol structures, see: Ferguson *et al.* (1995 ▶).
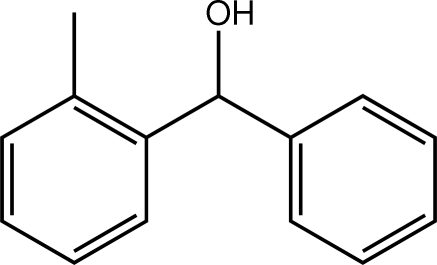


## Experimental

### 

#### Crystal data

C_14_H_14_O
*M* *_r_* = 198.25
Trigonal, 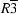

*a* = 23.013 (2) Å
*c* = 10.6067 (11) Å
*V* = 4864.8 (7) Å^3^
*Z* = 18
Mo *K*α radiation
μ = 0.08 mm^−1^
*T* = 100 K
0.40 × 0.35 × 0.30 mm

#### Data collection

Bruker SMART APEX CCD diffractometer
Absorption correction: multi-scan (*SADABS*; Sheldrick, 1996 ▶) *T*_min_ = 0.971, *T*_max_ = 0.978
6286 measured reflections
2475 independent reflections
2022 reflections with *I* > 2σ(*I*)
*R*_int_ = 0.026

#### Refinement

*R*[*F* ^2^ > 2σ(*F* ^2^)] = 0.045
*wR*(*F* ^2^) = 0.121
*S* = 1.08
2475 reflections
141 parameters
1 restraint
H atoms treated by a mixture of independent and constrained refinement
Δρ_max_ = 0.43 e Å^−3^
Δρ_min_ = −0.30 e Å^−3^

### 

Data collection: *APEX2* (Bruker, 2008 ▶); cell refinement: *SAINT* (Bruker, 2008 ▶); data reduction: *SAINT*; program(s) used to solve structure: *SHELXS97* (Sheldrick, 2008 ▶); program(s) used to refine structure: *SHELXL97* (Sheldrick, 2008 ▶); molecular graphics: *ORTEP-3* (Farrugia, 1997 ▶) and *DIAMOND* (Brandenburg, 2006 ▶); software used to prepare material for publication: *publCIF* (Westrip, 2010 ▶).

## Supplementary Material

Crystal structure: contains datablocks general, I. DOI: 10.1107/S1600536810029417/hb5570sup1.cif

Structure factors: contains datablocks I. DOI: 10.1107/S1600536810029417/hb5570Isup2.hkl

Additional supplementary materials:  crystallographic information; 3D view; checkCIF report

## Figures and Tables

**Table 1:** Hydrogen-bond geometry (Å, °)

*D*—H⋯*A*	*D*—H	H⋯*A*	*D*⋯*A*	*D*—H⋯*A*
O1—H1⋯O1^i^	0.85 (1)	1.85 (1)	2.6967 (10)	174 (2)

Symmetry code: (i) 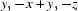.

**Fig. 1.**
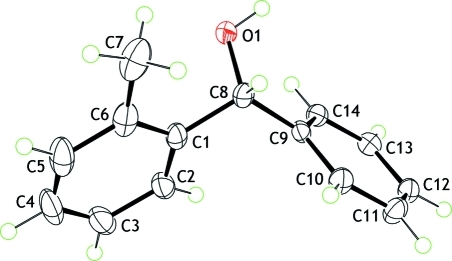
The molecular structure of (I) showing displacement ellipsoids at the 50% probability level.

**Fig. 2.**
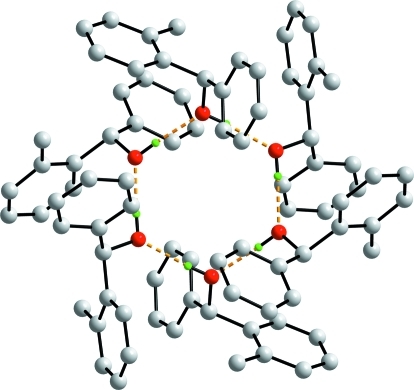
A hexameric aggregate mediated by O–H···O hydrogen bonds (orange dashed lines) in (I). Non-participating H atoms have been omitted.

**Fig. 3.**
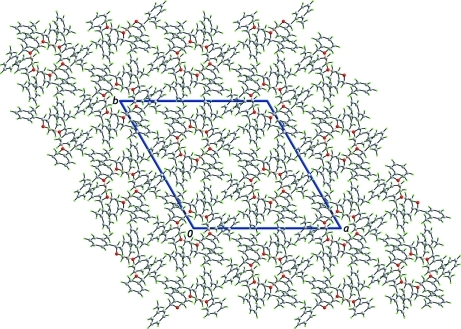
The unit-cell contents shown in projection down the c axis in (I). The O–H···O hydrogen bonding is shown as orange dashed lines.

## supplementary crystallographic information

### Comment

Benzhydrols are widely used as intermediates for the synthesis of pharmaceuticals (Ohkuma *et al.*, 2000), including drugs such as diphenhydramine, orphenadrine, diphenidol and phenyltoloxamine. The crystal structures and hydrogen bonding in some diphenylmethanols have been reported (Ferguson *et al.*, 1995). The title compound, phenyl-*o*-tolyl-methanol, (I), is a derivative of diphenylmethanol and it has use in the perfume and pharmaceutical industries (Meguro *et al.*, 1985).

The molecular structure of (I), Fig. 1, features a tertiary C7 atom connected to benzene and *o*-tolyl rings. With reference to the benzene ring, the O1 atom is nearly co-planar as seen in the O1–C8–C9–C14 torsion angle of -17.47 (17) °. By contrast, the *o*-tolyl group is almost orthogonal as seen in the C1–C8–C9–C10 torsion angle of -80.12 (15) °; the dihedral angle formed between the two least-squares planes is 87.78 (8) °. While lying to the same side of the molecule, the hydroxy and methyl groups are *gauche*.

The crystal packing is dominated by O–H···O hydrogen bonding, Table 1. Almost planar 12-membered rings comprising six O–H···O hydrogen bonds are found, each disposed about a site of symmetry, 3, Fig. 2. The hexameric aggregates stack in columns aligned along the *c* axis, Fig. 3.

### Experimental

The title compound was obtained as a gift from R. L. Fine Chemicals, Bangalore, India.  Colourless blocks of (I) were obtained by the slow evaporation of its acetonitrile solution; m.pt. 369–372 K.

### Refinement

Carbon-bound H-atoms were placed in calculated positions (C—H 0.95 to 1.00 Å) and were included in the refinement in the riding model approximation, with *U*_iso_(H) set to 1.2 to 1.5*U*_equiv_(C). The O-bound H-atom was located in a difference Fourier map, and was refined with a distance restraint of O–H 0.84±0.01 Å; the *U_iso_* value was freely refined

### Crystal data

**Table:**

C_14_H_14_O	*D*_x_ = 1.218 Mg m^−^^3^
*M**_r_* = 198.25	Mo *K*α radiation, λ = 0.71073 Å
Trigonal, *R*3	Cell parameters from 2551 reflections
Hall symbol: -R 3	θ = 2.8–28.3°
*a* = 23.013 (2) Å	µ = 0.08 mm^−^^1^
*c* = 10.6067 (11) Å	*T* = 100 K
*V* = 4864.8 (7) Å^3^	Block, colourless
*Z* = 18	0.40 × 0.35 × 0.30 mm
*F*(000) = 1908	

### Data collection

**Table:**

Bruker SMART APEX CCD diffractometer	2475 independent reflections
Radiation source: fine-focus sealed tube	2022 reflections with *I* > 2σ(*I*)
graphite	*R*_int_ = 0.026
ω scans	θ_max_ = 27.5°, θ_min_ = 1.8°
Absorption correction: multi-scan (*SADABS*; Sheldrick, 1996)	*h* = −28→28
*T*_min_ = 0.971, *T*_max_ = 0.978	*k* = −29→14
6286 measured reflections	*l* = −13→13

### Refinement

**Table:**

Refinement on *F*^2^	Primary atom site location: structure-invariant direct methods
Least-squares matrix: full	Secondary atom site location: difference Fourier map
*R*[*F*^2^ > 2σ(*F*^2^)] = 0.045	Hydrogen site location: inferred from neighbouring sites
*wR*(*F*^2^) = 0.121	H atoms treated by a mixture of independent and constrained refinement
*S* = 1.08	*w* = 1/[σ^2^(*F*_o_^2^) + (0.0533*P*)^2^ + 4.2609*P*] where *P* = (*F*_o_^2^ + 2*F*_c_^2^)/3
2475 reflections	(Δ/σ)_max_ < 0.001
141 parameters	Δρ_max_ = 0.43 e Å^−^^3^
1 restraint	Δρ_min_ = −0.30 e Å^−^^3^

### Special details

**Table:**

Geometry. All e.s.d.'s (except the e.s.d. in the dihedral angle between two l.s. planes) are estimated using the full covariance matrix. The cell e.s.d.'s are taken into account individually in the estimation of e.s.d.'s in distances, angles and torsion angles; correlations between e.s.d.'s in cell parameters are only used when they are defined by crystal symmetry. An approximate (isotropic) treatment of cell e.s.d.'s is used for estimating e.s.d.'s involving l.s. planes.
Refinement. Refinement of *F*^2^ against ALL reflections. The weighted *R*-factor *wR* and goodness of fit *S* are based on *F*^2^, conventional *R*-factors *R* are based on *F*, with *F* set to zero for negative *F*^2^. The threshold expression of *F*^2^ > σ(*F*^2^) is used only for calculating *R*-factors(gt) *etc*. and is not relevant to the choice of reflections for refinement. *R*-factors based on *F*^2^ are statistically about twice as large as those based on *F*, and *R*- factors based on ALL data will be even larger.

### Fractional atomic coordinates and isotropic or equivalent isotropic displacement parameters (Å^2^)

**Table:**

	*x*	*y*	*z*	*U*_iso_*/*U*_eq_	
O1	0.13170 (5)	0.08915 (5)	0.01453 (9)	0.0229 (2)	
H1	0.1201 (9)	0.0483 (5)	0.0010 (18)	0.043 (5)*	
C1	0.20865 (7)	0.19098 (7)	0.11187 (14)	0.0237 (3)	
C2	0.18864 (8)	0.21209 (7)	0.21715 (15)	0.0306 (3)	
H2	0.1637	0.1808	0.2816	0.037*	
C3	0.20471 (9)	0.27864 (8)	0.22928 (18)	0.0417 (4)	
H3	0.1911	0.2930	0.3018	0.050*	
C4	0.24081 (9)	0.32372 (8)	0.1346 (2)	0.0495 (5)	
H4	0.2523	0.3694	0.1423	0.059*	
C5	0.26018 (8)	0.30288 (8)	0.0295 (2)	0.0433 (5)	
H5	0.2847	0.3344	−0.0349	0.052*	
C6	0.24463 (7)	0.23669 (7)	0.01529 (16)	0.0321 (4)	
C7	0.26458 (8)	0.21578 (9)	−0.10289 (17)	0.0422 (4)	
H7A	0.2878	0.2545	−0.1591	0.063*	
H7B	0.2244	0.1806	−0.1450	0.063*	
H7C	0.2946	0.1984	−0.0819	0.063*	
C8	0.18897 (6)	0.11780 (6)	0.09612 (12)	0.0206 (3)	
H8	0.2264	0.1162	0.0515	0.025*	
C9	0.17524 (6)	0.07865 (6)	0.21775 (12)	0.0183 (3)	
C10	0.22851 (7)	0.08429 (7)	0.28925 (13)	0.0247 (3)	
H10	0.2733	0.1148	0.2642	0.030*	
C11	0.21660 (7)	0.04561 (7)	0.39708 (14)	0.0276 (3)	
H11	0.2533	0.0500	0.4457	0.033*	
C12	0.15144 (7)	0.00071 (7)	0.43395 (13)	0.0247 (3)	
H12	0.1433	−0.0263	0.5069	0.030*	
C13	0.09838 (7)	−0.00461 (7)	0.36404 (13)	0.0229 (3)	
H13	0.0536	−0.0350	0.3894	0.027*	
C14	0.11032 (7)	0.03426 (6)	0.25682 (13)	0.0207 (3)	
H14	0.0735	0.0304	0.2095	0.025*	

### Atomic displacement parameters (Å^2^)

**Table:**

	*U*^11^	*U*^22^	*U*^33^	*U*^12^	*U*^13^	*U*^23^
O1	0.0260 (5)	0.0200 (5)	0.0247 (5)	0.0130 (4)	−0.0060 (4)	−0.0017 (4)
C1	0.0180 (6)	0.0186 (6)	0.0340 (8)	0.0087 (5)	−0.0060 (5)	0.0017 (5)
C2	0.0317 (8)	0.0235 (7)	0.0397 (9)	0.0161 (6)	−0.0096 (6)	−0.0041 (6)
C3	0.0449 (10)	0.0297 (8)	0.0577 (11)	0.0241 (8)	−0.0221 (8)	−0.0149 (8)
C4	0.0386 (10)	0.0164 (7)	0.0895 (15)	0.0106 (7)	−0.0297 (10)	−0.0038 (8)
C5	0.0261 (8)	0.0226 (8)	0.0717 (13)	0.0051 (6)	−0.0108 (8)	0.0127 (8)
C6	0.0162 (6)	0.0257 (7)	0.0487 (10)	0.0062 (6)	−0.0050 (6)	0.0106 (7)
C7	0.0275 (8)	0.0477 (10)	0.0491 (10)	0.0171 (8)	0.0090 (7)	0.0230 (8)
C8	0.0190 (6)	0.0213 (6)	0.0239 (7)	0.0118 (5)	−0.0006 (5)	0.0022 (5)
C9	0.0205 (6)	0.0158 (6)	0.0209 (6)	0.0107 (5)	−0.0001 (5)	−0.0007 (5)
C10	0.0188 (6)	0.0233 (7)	0.0301 (7)	0.0091 (5)	−0.0017 (5)	0.0023 (5)
C11	0.0246 (7)	0.0302 (8)	0.0289 (8)	0.0144 (6)	−0.0055 (6)	0.0027 (6)
C12	0.0301 (7)	0.0228 (7)	0.0234 (7)	0.0148 (6)	−0.0001 (5)	0.0027 (5)
C13	0.0220 (7)	0.0217 (6)	0.0237 (7)	0.0100 (5)	0.0020 (5)	−0.0008 (5)
C14	0.0193 (6)	0.0218 (6)	0.0230 (7)	0.0118 (5)	−0.0014 (5)	−0.0016 (5)

### Geometric parameters (Å, °)

**Table:**

O1—C8	1.4323 (16)	C7—H7B	0.9800
O1—H1	0.852 (9)	C7—H7C	0.9800
C1—C2	1.385 (2)	C8—C9	1.5137 (18)
C1—C6	1.404 (2)	C8—H8	1.0000
C1—C8	1.5188 (18)	C9—C14	1.3862 (18)
C2—C3	1.390 (2)	C9—C10	1.3911 (18)
C2—H2	0.9500	C10—C11	1.390 (2)
C3—C4	1.383 (3)	C10—H10	0.9500
C3—H3	0.9500	C11—C12	1.386 (2)
C4—C5	1.373 (3)	C11—H11	0.9500
C4—H4	0.9500	C12—C13	1.3806 (19)
C5—C6	1.388 (2)	C12—H12	0.9500
C5—H5	0.9500	C13—C14	1.3868 (19)
C6—C7	1.495 (3)	C13—H13	0.9500
C7—H7A	0.9800	C14—H14	0.9500
			
C8—O1—H1	108.5 (13)	O1—C8—C9	111.77 (10)
C2—C1—C6	120.03 (13)	O1—C8—C1	105.81 (10)
C2—C1—C8	120.69 (13)	C9—C8—C1	115.10 (11)
C6—C1—C8	119.20 (13)	O1—C8—H8	108.0
C1—C2—C3	120.59 (16)	C9—C8—H8	108.0
C1—C2—H2	119.7	C1—C8—H8	108.0
C3—C2—H2	119.7	C14—C9—C10	118.73 (12)
C4—C3—C2	119.23 (18)	C14—C9—C8	121.37 (12)
C4—C3—H3	120.4	C10—C9—C8	119.81 (11)
C2—C3—H3	120.4	C11—C10—C9	120.43 (13)
C5—C4—C3	120.38 (15)	C11—C10—H10	119.8
C5—C4—H4	119.8	C9—C10—H10	119.8
C3—C4—H4	119.8	C12—C11—C10	120.20 (13)
C4—C5—C6	121.39 (17)	C12—C11—H11	119.9
C4—C5—H5	119.3	C10—C11—H11	119.9
C6—C5—H5	119.3	C13—C12—C11	119.60 (13)
C5—C6—C1	118.37 (16)	C13—C12—H12	120.2
C5—C6—C7	119.48 (15)	C11—C12—H12	120.2
C1—C6—C7	122.12 (14)	C12—C13—C14	120.14 (13)
C6—C7—H7A	109.5	C12—C13—H13	119.9
C6—C7—H7B	109.5	C14—C13—H13	119.9
H7A—C7—H7B	109.5	C9—C14—C13	120.89 (12)
C6—C7—H7C	109.5	C9—C14—H14	119.6
H7A—C7—H7C	109.5	C13—C14—H14	119.6
H7B—C7—H7C	109.5		
			
C6—C1—C2—C3	−1.0 (2)	C6—C1—C8—C9	157.52 (12)
C8—C1—C2—C3	−177.73 (13)	O1—C8—C9—C14	−17.47 (17)
C1—C2—C3—C4	0.3 (2)	C1—C8—C9—C14	103.27 (14)
C2—C3—C4—C5	0.4 (2)	O1—C8—C9—C10	159.14 (12)
C3—C4—C5—C6	−0.3 (2)	C1—C8—C9—C10	−80.12 (15)
C4—C5—C6—C1	−0.4 (2)	C14—C9—C10—C11	0.6 (2)
C4—C5—C6—C7	177.76 (15)	C8—C9—C10—C11	−176.11 (12)
C2—C1—C6—C5	1.0 (2)	C9—C10—C11—C12	0.4 (2)
C8—C1—C6—C5	177.84 (13)	C10—C11—C12—C13	−1.0 (2)
C2—C1—C6—C7	−177.07 (14)	C11—C12—C13—C14	0.7 (2)
C8—C1—C6—C7	−0.3 (2)	C10—C9—C14—C13	−0.92 (19)
C2—C1—C8—O1	98.25 (14)	C8—C9—C14—C13	175.72 (12)
C6—C1—C8—O1	−78.54 (14)	C12—C13—C14—C9	0.3 (2)
C2—C1—C8—C9	−25.70 (17)		

### Hydrogen-bond geometry (Å, °)

**Table:**

*D*—H···*A*	*D*—H	H···*A*	*D*···*A*	*D*—H···*A*
O1—H1···O1^i^	0.85 (1)	1.85 (1)	2.6967 (10)	174 (2)

Symmetry codes: (i) *y*, −*x*+*y*, −*z*.
